# In-hospital recurrence in a Chinese large cohort with acute ischemic stroke

**DOI:** 10.1038/s41598-019-51277-8

**Published:** 2019-10-18

**Authors:** Fan Yu, Xiaolu Liu, Qiong Yang, Yu Fu, Dongsheng Fan

**Affiliations:** 1 Department of Neurology, Peking University Third Hospital, Beijing, China; 2 Department of Neurology, Yulin No.2 Hospital, Yulin, Shaanxi Province China; 3 Key Laboratory for Neuroscience, National Health Commission/Ministry of Education, Peking University, Beijing, China

**Keywords:** Neurology, Stroke

## Abstract

Acute ischemic stroke (AIS) has a high risk of recurrence, particularly in the early stage. The purpose of this study was to assess the frequency and risk factors of in-hospital recurrence in patients with AIS in China. A retrospective analysis was performed of all of the patients with new-onset AIS who were hospitalized in the past three years. Recurrence was defined as a new stroke event, with an interval between the primary and recurrent events greater than 24 hours; other potential causes of neurological deterioration were excluded. The risk factors for recurrence were analyzed using univariate and logistic regression analyses. A total of 1,021 patients were included in this study with a median length of stay of 14 days (interquartile range,11–18). In-hospital recurrence occurred in 58 cases (5.68%), primarily during the first five days of hospitalization. In-hospital recurrence significantly prolonged the hospital stay (P < 0.001), and the in-hospital mortality was also significantly increased (P = 0.006). The independent risk factors for in-hospital recurrence included large artery atherosclerosis, urinary or respiratory infection and abnormal blood glucose, whereas recurrence was less likely to occur in the patients with aphasia. Our study showed that the patients with AIS had a high rate of in-hospital recurrence, and the recurrence mainly occurred in the first five days of the hospital stay. In-hospital recurrence resulted in a prolonged hospital stay and a higher in-hospital mortality rate.

## Introduction

In the acute phase of ischemic stroke, the patient’s condition is unstable. Even during hospitalization, in which the sufficient secondary prevention be provided, recurrence may still occur in some stroke patients. A multicenter retrospective study by Erdur *et al*. showed that the in-hospital recurrence rate of ischemic stroke in patients with acute ischemic stroke (AIS) was 0.8% in Germany, and the independent risk factors of recurrence included a history of transient ischemic attack (TIA) and severe symptomatic carotid artery stenosis, as well as other determined causes^1^. So far, information on the incidence rate and risk factors of in-hospital recurrence of patients with AIS in China is lacking. This study used a retrospective analysis of the clinical data of the AIS patients hospitalized in our center to preliminarily investigate the frequency, time profile, and risk factors affecting the adverse outcomes of in-hospital recurrence in Chinese patients with AIS.

## Materials and Methods

### Study setting and sample

This was based on a cohort study including patients with acute ischemic stroke (defined as ≤14 d) admitted to Peking University Third Hospital in the past 3 years. The study was approved by the institutional ethics committee of Peking University Third Hospital and all methods were performed in accordance with the relevant guidelines and regulations. Informed consents were obtained from all patients.

The diagnoses of AIS were confirmed by a neurologist based on the WHO definition and the combination of the medical history, clinical manifestations and imaging study (CT or MRI)^2^.

The following data were collected and recorded: gender; age; initial onset of symptoms (aphasia, limb weakness, and unconsciousness); medical history (a history of hypertension, diabetes, hyperlipidemia, stroke or TIA); the severity when enrolled in this study, which was evaluated using the National Institutes of Health Stroke Scale (NIHSS); and other auxiliary examinations including carotid artery ultrasound, head CT, MRI, CTA, MRA, DSA, echocardiography, and Holter dynamic electrocardiogram with the corresponding percentages. Simultaneously, the detailed treatment for the patients within 24 hours after admission was recorded, including intravenous or intra-arterial thrombolysis, antiplatelet therapy, anticoagulation therapy, blood pressure control, blood glucose control and statin therapy. According to the Trial of Org10172 in Acute Stroke Treatment (TOAST), the etiology of ischemic stroke was divided into five categories including large artery atherosclerosis (LAA), cardiac, small vessel disease (SVD), other clear causes, and other unknown causes^3^. The medical records of the patients were carefully evaluated for the presence of lung or urinary tract infections based on documented diagnosis and beginning of antimicrobial therapy, whereas the following clinical indicators of the patients after admission were extracted: the first systolic blood pressure (SBP) after admission, all SBP values within 24 hours, the calculated 24-hour average blood pressure, the variation coefficient of blood pressure, blood lipids (cholesterol, triglycerides, low-density lipoprotein, and high-density lipoprotein), blood glucose (glycated hemoglobin, fasting blood glucose, blood glucose level within 24 hours to determine whether the glucose metabolism was abnormal), homocysteine, and hypersensitive C-reactive protein. The abnormal glucose levels defined fasting blood glucose ≤3.3 mmol/l or ≥10 mmol/l.

The primary endpoint was recurrent ischemic event in the period of hospitalization, in which a recurrent stroke was defined as the following: a new stroke event, with stable interval nerve function for more than 24 hours between the primary onset and the recurrence. Other potential causes for neurological deterioration were excluded^4^. When the new ischemic event was located at the same vascular region as the primary event and was significantly associated with the new neurological symptoms, it was considered to be a recurrent stroke. The progression or aggravation of stroke due to other causes (such as hemorrhagic transformation, edema, etc.) and its corresponding neurological deterioration were not considered recurrent stroke.

### Data analysis

The continuous variables in our study were not normally distributed, so they were represented as the median and interquartile range, whereas the categorical variables were represented as percentages. The differences of the continuous variables were compared using the Mann-Whitney U test. The X^2^ test was used to test the dichotomous variables. A univariate analysis and logistic regression were applied. If a variable showed an association of P < 0.05 in the univariate comparison and generated ≥1 events, it was included in the multivariate model. The results of the multivariate regression analysis were represented using OR values and 95% confidence interval, and differences with P < 0.05 were considered statistically significant. The data analysis was performed using SPSS (17.0) software.

## Results

**Figure 1:**
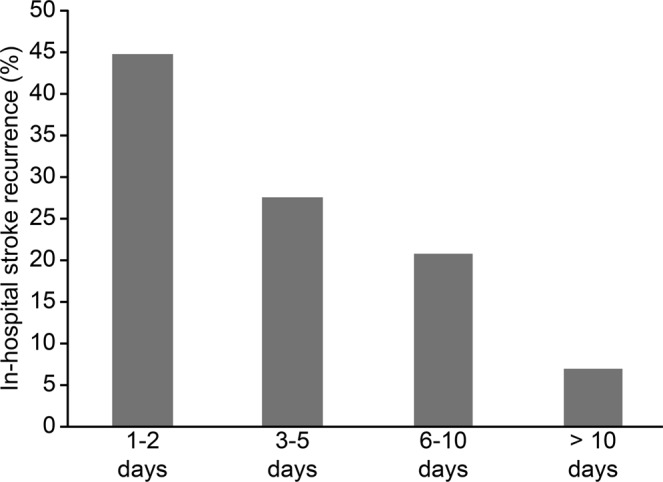
The time profile of in-hospital stroke recurrence.

This study included 1,021 patients, of whom 73.4% were male (median age, 64 years; interquartile range, 55–75; median NIHSS 2; interquartile range, 1–5; median length of hospital stay, 14 days; interquartile range, 11–18). Among all of the patients, in-hospital recurrence occurred in 58 cases, with a recurrence rate of 5.68%. Recurrent stroke mainly occurred in the first five days of hospitalization, accounting for approximately 73% of all recurrent strokes (Fig. 1). The length of hospital stay for the patients with in-hospital recurrence was significantly prolonged compared to the patients without recurrence, and the mortality was also significantly increased (Table 1).

**Table 1:** Comparison of hospital stay and in-hospital mortality between the stroke patients in the recurrence group and the non-recurrence group.

	Total N = 1021	Recurrence N = 58	Non-recurrence N = 963	P value
Length of hospital stay (days)	14 (11–18)	18 (14–22)	14 (11–17)	<0.000
In-hospital mortality (%)	1.3	5.2	1.0	0.006

### Comparison of the baseline between the two groups

A comparison of the baselines for all cases and the two groups of patients are shown in Table 2. According to TOAST classification, the patients with LAA accounted for 43.7% of stroke patients, whereas those with cardiac, SVD, other determined etiology, and undetermined etiology comprised 8.1%, 24.1%, 2.1%, and 21.7% of stroke patients, respectively^5,6^.

**Table 2:** Comparison of the baseline characteristics of the patients with and without recurrent stroke.

	Total N = 1021	Recurrence N = 58	Non-recurrence N = 963	P value
**Demographic characteristics**				
Age (years)	64 (55–75)	68 (60–76)	64 (55–74)	0.122
Gender (male, %)	749 (73.4%)	46 (79.3%)	703 (73.0%)	0.291
**Medical history**				
Hypertension (%)	697 (68.3%)	41 (70.7%)	656 (68.1%)	0.983
Diabetes (%)	351 (34.4%)	27 (46.6%)	324 (33.7%)	0.044
Hyperlipidemia (%)	219 (21.5%)	18 (31.0%)	201 (20.9%)	0.067
History of stroke (%)	245 (24.0%)	11 (19.0%)	234 (24.3%)	0.356
History of TIA (%)	66 (6.5%)	8 (13.8%)	58 (6.0%)	0.019
Atrial fibrillation (%)	93 (9.1%)	7 (12.1%)	86 (8.4%)	0.420
**Clinical characteristics**				
Limb weakness (%)	710 (69.5%)	43 (74.2%)	667 (65.3%)	0.433
Aphasia (%)	360 (35.3%)	12 (20.7%)	348 (36.1%)	0.017
Consciousness (%)	28 (2.7%)	0 (0%)	28 (2.9%)	0.188
NIHSS score	2 (1–5)	3 (1–5)	2 (1–2)3.46 ± 3.63	0.745
**Etiology**				
LAA (%)	446 (43.7%)	40 (69.0%)	406 (42.2%)	<0.000
CE (%)	83 (8.1%)	6 (10.4%)	77 (8.0%)	0.525
SVD (%)	246 (24.1%)	0 (0%)	246 (25.6%)	<0.000
OD (%)	21 (2.1%)	3 (5.2%)	18 (1.9%)	0.085
UD (%)	222 (21.7%)	9 (15.5%)	213 (22.1%)	0.237

### Comparison of the diagnosis and treatment measures between the two groups

Measures of diagnosis and treatment are presented in Table 3. In our study, all patients underwent a CT scan or an MRI, and 20.7% of patients received both of them. Only 6.9% the patients with recurrent stroke and 8% of the patients with non-recurrent stroke underwent Holter dynamic electrocardiogram^7^. Only seven patients underwent endovascular treatment. The diagnosis and treatment measures for the patients with recurrence and non-recurrence were not significantly different.

**Table 3:** Comparison of the diagnosis and treatment measures between the two groups of patients.

	Total N = 1021	Recurrence N = 58	Non-recurrence N = 963	P value
Head CT (%)	855 (83.74)	52 (89.66%)	803 (83.39%)	0.209
Head MRI (%)	979 (95.89%)	55 (94.83%)	924 (95.95%)	0.676
Head MRA, CTA, DSA (%)	907 (88.83%)	50 (86.21%)	857 (88.99%)	0.513
Cervical vascular ultrasound (%)	938 (91.87%)	55 (94.83%)	882 (91.59%)	0.179
Echocardiography (%)	952 (93.24%)	54 (93.10%)	898 (93.25%)	0.965
Dynamic electrocardiogram (%)	81 (7.93%)	4 (6.90%)	77 (8.00%)	0.764
Intravenous thrombolysis (%)	92 (9.01%)	4 (6.90%)	88 (9.14%)	0.812
Antiplatelet (%)	984 (96.38%)	55 (94.83%)	929 (96.47%)	0.462
Anticoagulant (%)	35 (3.43%)	4 (6.90%)	31 (3.22%)	0.132
Antihypertensive drug (%)	492 (48.19%)	27 (46.55%)	465 (48.29%)	0.797
Blood glucose reduction (%)	288 (28.21%)	21 (36.21%)	267 (27.73%)	0.163
Statins (%)	933 (91.38%)	55 (94.83%)	878 (85.99%)	0.336
Endovascular treatment	7 (0.69%)	2 (3.44%)	5 (0.52%)	0.055

### Comparison of the tested indicators and relevant blood pressure during the hospitalization between the two groups

As shown in Table 4, the values of homocysteine, ultra-sensitivity C-reactive protein, total cholesterol, triglycerides, low-density lipoprotein, high-density lipoprotein, the ratio of low density lipoprotein and high-density lipoprotein, and the blood pressure variation coefficient were not significantly different between the two groups of patients. However, the values of fasting blood glucose, glycated hemoglobin, the percentages of abnormal blood glucose, and the values of basic SBP and 24-hour average SBP for the patients in the recurrence group were higher than those with no recurrence.

**Table 4:** Comparison of the serology results and blood pressure between the two groups of patients.

	Total N = 1021	Recurrence N = 58	Non-recurrence N = 963	P value
Hcy	15.17 (12.31–19.37)	14.97 (12.38–18.25)	15.21 (12.31–19.39)	0.909
CRP	2.03 (0.99–5.99)	2.01 (1.17–8.32)	2.03 (0.98–5.97)	0.338
CHL	4.16 (3.50–4.86)	3.71 (3.31–5.06)	4.19 (3.53–4.84)	0.293
TG	1.38 (1.04–1.90)	1.38 (1.09–1.80)	1.38 (1.04–1.92)	0.950
LDLC	2.46 (1.90–3.08)	2.19 (1.77–3.09)	2.47 (1.91–3.08)	0.320
HDLC	0.95 (0.83–1.12)	0.97 (0.86–1.12)	0.95 (0.83–1.12)	0.640
LDLC/HDLC	2.51 (1.95–3.25)	2.36 (1.95–3.05)	2.53 (1.94–3.26)	0.280
FBG	5.10 (4.60–6.30)	5.45 (4.80–7.33)	5.10 (4.60–6.23)	0.019
HbA1c	5.80 (5.40–6.80)	6.05 (5.60–7.63)	5.80 (5.40–6.70)	0.017
Abnormal blood glucose	83 (8.13%)	14 (24.14%)	69 (7.17%)	<0.000
Blood pressure at admission	140 (130–154)	146 (134–160)	140 (130–153)	0.017
Average blood pressure	138 (129–147)	143 (134–153)	138 (129–148)	0.013
Blood pressure variation coefficient	0.070 (0.481–0.095)	0.077 (0.057–0.106)	0.069 (0.048–0.094)	0.052

### Comparison of the risk factors of stroke recurrence between the two groups

According to the univariate analysis, in the 58 patients with in-hospital recurrent stroke (5.68%), the patients with a history of diabetes, a history of TIA, the TOAST classification of LAA, lung or urinary tract infection between the two strokes, high glycated hemoglobin, higher FBG, abnormal blood glucose, higher basic SBP, and higher average SBP values showed a high frequency of recurrent stroke. However, the patients with aphasia showed a low frequency of recurrent stroke, and no patients with the TOAST classification of SVD experienced recurrent stroke. The multivariate regression analysis showed that the etiology of LAA, urinary tract and lung infection between the two strokes, and abnormal blood glucose values within 24 hours after admission still increased the risk of recurrent stroke, whereas the stroke recurrence risk was low in the patients with aphasia (Table 5). A history of TIA, a history of diabetes, the value of glycosylated hemoglobin and FBG, the basic blood pressure value and the 24-hour average blood pressure showed no significant correlation with recurrent stroke in the multivariate regression analysis.

**Table 5:** Multivariate regression analysis to identify the independent risk factors for recurrent stroke.

	OR	95% CI		P value
Large artery atherosclerosis	2.832	1.547	5.184	0.001
Infection	3.72	1.709	8.104	0.001
Abnormal blood glucose	5.888	2.091	16.581	0.001
Aphasia	0.408	0.206	0.808	0.010

## Discussion

The recurrence rate within one week after a stroke varies greatly indifferent studies, ranging from 1.0 to 6.2%^8–16^. This is the first study to investigate the in-hospital recurrence in AIS patients in China. The results showed that the incidence rate of in-hospital recurrent stroke was approximately 5.7% in Chinese patients, which is much higher than the recurrent stroke rate of only 0.8% in Germany counterpart hospitals^1^. This study also noted that in-hospital recurrent stroke mainly occurred in the first five days of hospitalization, accounting for approximately 73% of all recurrences. As expected, the hospitalization of the patients in the recurrence group was significantly longer and the in-hospital mortality was also significantly higher, which supports the idea that early recurrence of stroke is often accompanied by adverse outcomes.

The high in-hospital recurrence rate of stroke in this study was presumably caused by the following factors: (1) the length of hospital stay for the stroke patients in China was significantly long, with a median length of hospitalization in the present study of 14 days, whereas the median length of hospitalization in Germany was only five days; (2) according to TOAST classification, in this study, the incidence of LAA was significantly higher than that in the report of Erdur (43.7 vs. 2.9%)^1^, and LAA had been known to be an independent risk factor for stroke recurrence; and (3) in the present study, the rates of endovascular treatment for the patients with significant stenosis caused by LAA and anticoagulation therapy for the cardiac patients were low, and “Early use of Existing Preventive Strategies for Stroke” (EXPRESS) found that early initiation of antithrombotic therapy, anticoagulation, statins therapy, and blood pressure control, as well as secondary prevention measures including endarterectomy for symptomatic internal carotid stenosis in the early stage, could reduce the risk of recurrent stroke by 80%^17^. Another study also showed that endarterectomy as early as possible for patients with symptomatic carotid internal artery stenosis could greatly benefit the prognosis^18^. Carotid revascularization has been recommended as the maximally beneficial treatment for stroke prevention in patients with recently symptomatic carotid stenosis. Because of the periprocedural risk, the appropriate timing for performing carotid endarterectomy within the first 14 d after the occurrence of the stroke remains controversial. A systematic review conducted by Savardekar *et al*.^19^ noted a changing paradigm towards early carotid surgery, specifically targeted within 48 h if the index event is TIA, and within 7 d if the index event is stroke.

In the risk factor analysis, the present study showed that the independent risk factors for stroke recurrence were the etiology of LAA, abnormal blood glucose, and lung or urinary tract infection between the two strokes. Conversely, the patients with SVD as the etiology had a low incidence of recurrence, and the associated aphasia symptom was often correlated with a low risk of recurrence. A history of TIA and an increased ratio of low-density and high-density lipoprotein cholesterol in the previous studies did not show a significant correlation with the in-hospital stroke recurrence in our study.

Similar to findings in other studies showing that abnormal glucose metabolism is an independent predictor of stroke recurrence^20–24^, this study also showed that blood glucose abnormality in the early stage after stroke was significantly related to in-hospital stroke recurrence. However, no significant difference was found in the multivariate analysis for a history of diabetes, glycated hemoglobin, and fasting blood glucose. This study found that urinary tractor or lung infection was a risk factor for in-hospital stroke recurrence. Many factors were associated with the occurrence of post-stroke infection, such as age, which was complicated by diabetes mellitus, being bedridden, unconsciousness, and invasive procedures, in which urinary tractor or lung infection is the main form of complicated infection after acute stroke^25^. The results of this study supported the idea that in-hospital infection would cause a prolonged hospital stay and higher stroke morbidity^26^. The mechanism of recurrent stroke was more likely to occur in the patients complicated with infection because the inflammation may lead to a hypercoagulable state, platelet activation, and endothelialinjury^27,28^. Therefore, clinicians should place a high emphasis on patients showing infection after stroke during hospitalization, and the guidelines also strongly recommend the use of antibiotics to control the infection as early as possible^29^.

This study confirmed the different outcomes in different subtypes of stroke. The cases with LAA showed a high recurrence rate, and those with SVD had a lower risk of early recurrence of stroke^1,12,30,31^. Because patients with LAA have a high risk of recurrence in the early stage, clinicians should perform an urgent assessment of blood vessels. Consistent with the study of Erdur *et al*.^1^, we also found that aphasia was associated with a low in-hospital recurrence of stroke. As a possible reason, patients with aphasia may have difficulties in expression, so the newly emerging neurological deficit might be ignored in clinical practice; thus, the recurrence could be undervalued. However, further investigation is required to determine whether there are other mechanisms.

When to initiate blood pressure control and to what extent blood pressure should be reduced after acute stroke remains uncertain. Our results showed that the values of SBP after admission and the average SBP of the patients in the recurrence group were higher than those of the patients without recurrence based on the univariate analysis; however, according to the multivariate analysis, the basic SBP, the average SBP, and variations in blood pressure within 24 hours were not independently associated with in-hospital stroke recurrence. The CATIS study^32^ showed that lowering the blood pressure within 48 hours after stroke did not reduce death and major disability within 14 days or at discharge. A study by Manning *et al*.^33^ also showed that the transient blood pressure variability was not correlated with death and disability within two weeks after the stroke. A Chinese National Stroke Registry (CNSR) study^34^ showed that the effect of hypertension on the recurrence of stroke within one year was related to the subtype of the stroke, which only affected the patients with the SVD subtype. Further study with a larger sample size is expected in the future to analyze whether the effect of blood pressure is related to the stroke subtype in early recurrence after stroke, particularly in-hospital recurrence. The main limitations of this study include that the data analysis was based on a retrospective design, and as a single-center study, the population baseline characteristics and treatment measures may not be a good representation of the situation throughout the region. In our center, the ration of male to female beds is 3:1, which leads to a high proportion males in our study.

## Conclusion

Even hospitalization with the sufficient secondary prevention, stroke recurrence remain high in China. Patients with LAA, infection and glucose abnormality are at higher risk, But the risk in patients with SVD is relatively lower.
