# Publisher Correction: In-hospital recurrence in a Chinese large cohort with acute ischemic stroke

**DOI:** 10.1038/s41598-019-54658-1

**Published:** 2019-11-29

**Authors:** Fan Yu, Xiaolu Liu, Qiong Yang, Yu Fu, Dongsheng Fan

**Affiliations:** 1 Department of Neurology, Peking University Third Hospital, Beijing, China; 2 Department of Neurology, Yulin No.2 Hospital, Yulin, Shaanxi Province China; 3 Key Laboratory for Neuroscience, National Health Commission/Ministry of Education, Peking University, Beijing, China

Correction to: *Scientific Reports* 10.1038/s41598-019-51277-8, published online 18 October 2019

The original version of this Article omitted an affiliation for Fan Yu. The correct affiliations for Fan Yu are listed below:

Department of Neurology, Peking University Third Hospital, Beijing, China

Department of Neurology, Yulin No.2 Hospital, Yulin, Shaanxi Province, China

This has now been corrected in the HTML and PDF versions of this Article.

